# Comparative Transcriptome Profiling of the Maize Primary, Crown and Seminal Root in Response to Salinity Stress

**DOI:** 10.1371/journal.pone.0121222

**Published:** 2015-03-24

**Authors:** Maolin Zhang, Xiangpei Kong, Xiangbo Xu, Cuiling Li, Huiyu Tian, Zhaojun Ding

**Affiliations:** 1 The Key Laboratory of Plant Cell Engineering and Germplasm Innovation, College of Life Sciences, Shandong University, Jinan, 250100, Shandong, China; 2 Maize Institute, Shandong Academy of Agricultural Sciences/National Maize Improvement Sub-Center/National Engineering Laboratory for Wheat and Maize, Jinan, 250000, Shandong, China; Estación Experimental del Zaidín (CSIC), SPAIN

## Abstract

Soil salinity is a major constraint to crop growth and yield. The primary and lateral roots of *Arabidopsis thaliana* are known to respond differentially to a number of environmental stresses, including salinity. Although the maize root system as a whole is known to be sensitive to salinity, whether or not different structural root systems show differential growth responses to salinity stress has not yet been investigated. The maize primary root (PR) was more tolerant of salinity stress than either the crown root (CR) or the seminal root (SR). To understand the molecular mechanism of these differential growth responses, RNA-Seq analysis was conducted on cDNA prepared from the PR, CR and SR of plants either non-stressed or exposed to 100 mM NaCl for 24 h. A set of 444 genes were shown to be regulated by salinity stress, and the transcription pattern of a number of genes associated with the plant salinity stress response differed markedly between the various types of root. The pattern of transcription of the salinity-regulated genes was shown to be very diverse in the various root types. The differential transcription of these genes such as transcription factors, and the accumulation of compatible solutes such as soluble sugars probably underlie the differential growth responses to salinity stress of the three types of roots in maize.

## Introduction

Plants are exposed to various environmental stresses during their life cycle, and soil salinity is one of the leading constraints to plant growth and productivity. Salinity stress involves a combination of both ionic and osmotic stress, and these induce a range of conditions, including membrane dysfunction, metabolic disorder and oxidative stress [1–3]. Transgenic experiments have shown that the constitutive expression of certain signaling pathway genes (in particular those encoding certain protein kinases and transcription factors) can have a positive effect on tolerance [4–8]. Salinity-regulated transcription factors (TFs) control the expression of a wide range of genes, and one of the most important pathways involved is the SOS (*Salt Overly Sensitive*) signaling pathway, which is activated by cytoplasmic Ca^2+^ and has a major regulatory role over ion homeostasis [9–11].

Salinity stress inhibits the growth of the *Arabidopsis thaliana* primary root by suppressing cell division and elongation. It has been claimed that it also induces agravitropic primary root (PR) growth by its effect on the auxin efflux carrier PIN2 [12, 13]. The effect of salinity stress on the lateral root (LR) is less straight forward. Osmotic stress, as induced by salinity, inhibits LR emergence, although this can be rescued by the administration of exogenous auxin [14, 15]. Zhao et al. have shown that mild ionic stress stimulates both the initiation and emergence of LR, and that LR emergence in the loss-of-function *sos1*, *2* and *3* mutants is reduced in response to ionic, but not to osmotic stress [16]. Salinity has a major effect on root growth and development, but most relevant studies have focused on the root as a whole, even though the suspicion is that different roots from the same plant may respond differentially to the same environmental stress. Duan et al. have shown that LR growth is more strongly suppressed by salinity than is that of the PR, and that this difference is associated with ABA signaling [17, 18]. The two types of root also have a different gravitropic response, mediated by the effect of PIN on the redistribution of auxin [19, 20]. According to Vidal et al. [21], the microRNA miR393, which is inducibled by nitrate, affects only LR growth. All these investigations suggest that the differential growth dynamics between primary and lateral roots in *Arabidopsi*s are crucial for plants to adapt to the ever-changing environmental conditions.

*A*. *thaliana* forms a taproot, comprising of a single embryonically initiated PR and post-embryonically initiated LRs, but maize has a typical fibrous root system comprising of more or less the same size of embryonically and post-embryonically initiated branch roots [22, 23]. In maize, the embryonically initiated roots consist of the PR and a variable number of seminal roots (SRs), which plays important roles during the early stages of plant development. The post-embryonically initiated roots are represented by a combination of LR and shoot-borne roots initiated from stem nodes; those which emerge above the surface are referred to as brace roots, and those which emerge below the surface are known as crown roots (CR). The post-embryonic root system is important for the physiology of the mature plant.

Till now, genetic studies have identified and characterized several specific root mutants in maize. The mutant *rtcs* forms no shoot-borne roots and the embryonic seminal roots and is a consequence of the loss-of-function of *RTCS*, which encodes a LATERAL ORGAN BOUNDARIES domain (LBD) protein [24, 25]. The LR mutant *rum1* is largely unable to initiate either SR or LR from the PR, and the mutated gene *RUM1* encodes a truncated *ZmIAA10* sequence, which interacts directly with the auxin response factors ZmARF25 and ZmARF34 [26].

Here, the growth response of the various types of the maize root system to salinity stress was characterized, and the molecular basis of the differential growth responses in response to salinity stress between PR, SR and CR was investigated by physiological analysis and transcriptome comparisons.

## Material and Methods

### Plant materials and salinity treatment

In the beginning, maize (*Zea mays* L cv. Chang 7–2) seeds were rolled into soggy filter papers for several days before primary roots (PR) elongated up to about 3 cm, and then the seedlings were grown for two weeks in 1/2 Hoagland’s solution (0.51 g/L KNO_3_, 0.82 g/L Ca(NO_3_)_2_, 0.49 g/L MgSO_4_·7H_2_O, 0.136 g/L KH_2_PO_4_, 0.6 ml/L FeSO_4_, 2.86 mg/L H_3_BO_3_, 1.81 mg/L MnCl_2_·4H_2_O, 0.08 mg/L CuSO_4_·5H_2_O, 0.22 mg/L ZnSO_4_·7H_2_O, 0.09 mg/L H_2_MoO_4_·4H_2_O) (pH 6.0) in a growth chamber held at 28°C/25°C (day/night) and a relative humidity of 60% under a 16 h photoperiod provided by 100 μmol m^-2^ s^-1^ of photosynthetically active radiation. The culture solution was renewed every two days. Early developmental stage of primary root (PR) (3 days after germination), early developmental stage of seminal root (SR) (7 days after germination), and early developmental stage of crown root (CR) (15 days after germination) with lengths of approximately 6 to 10 cm were selected for the experiments.

For salt treatment, 40 roots from 40 individual seedlings with lengths of approximately 6 to 10 cm for each root type were transferred to Hoagland’s solution containing either 0 or 100 mM NaCl for 24 h or 72 h, the root length was measured before and after the NaCl treatment. Each root type was analyzed in triplicate.

For RNA sequencing, PR, SR and CR with lengths of approximately 6 to 10 cm were exposed for 24 h to the same Hoagland’s solution containing either 0 or 100 mM NaCl. About 10–15 roots from 10–15 individual seedlings with lengths of 1 cm from root tip for each root type were chosen for this experiment. The harvested tissues were immediately frozen in liquid nitrogen and stored at -80°C.

### cDNA library preparation and sequencing

Library construction and sequencing were performed according to the method described previously [27]. Total RNA was extracted from the root samples using the TRIzol reagent (Invitrogen, Carlsbad, CA, USA). The cDNA first strand was generated using reverse transcriptase and random primers. The resulting cDNA libraries were sequenced using an Illumina HiSeq 2000 platform at the Beijing Genomics Institute (Shenzhen, China). Sequencing data is available in the GEO Gene Expression Omnibus (GEO) database under accession number GSE53995.

### Assessment of differential gene transcription

Raw reads were filtered to remove low quality reads, according to the following procedure: 1) Remove reads with adaptor sequences. 2) Remove reads in which the percentage of unknown bases (N) is greater than 10%. 3) Remove low quality reads. If the percentage of the low quality base (base with quality value ≤ 5) is greater than 50% in a read, we define this read as low quality. The resulting set of reads was aligned with the maize cv. B73 RefGen_V2 genomic DNA sequence using SOAP 2.21 software, and related to known genes by a BLAST (BLAST 2.2.23) analysis (http://blast.ncbi.nlm.nih.gov/Blast.cgi). Transcript abundance was calculated using the RPKM method (reads per kb per million reads) [28] and the formula is shown as follows:

$$RPKM=\frac{10^{6}C}{NL/10^{3}}$$

where C is number of reads that uniquely aligned to gene X, N is total number of reads that uniquely aligned to all genes, and L is number of bases of gene X.

The assignment of differential transcription relied on the probability (*p*) value and the false discovery rate (FDR) [29]. The former corresponds to a differential gene transcription test, while the latter is used to determine the threshold *p*-value. The thresholds applied were FDR ≤ 0.001 and the absolute value of log_2_ (treatment/control) ≥ 1 [30].

All *p*-values were determined according to the following formula:

p(y|x)=(N2N1)y(x+y)!x!y!(1+N2N1)(x+y+1)

N_1_ and N_2_ denotes the total number of clean tags in two compared libraries, respectively, while x and y represents the clean tags mapping to gene X.

Functional classification of differentially transcribed genes (DTGs) exploited the GO (Gene Ontology) (http://www.geneontology.org/) and KEGG (Kyoto Encyclopedia of Genes and Genomes) (http://www.genome.jp/kegg/) databases. For GO analysis, annotating the results that from BLAST (-p blastx-e 1e-5-m 7) sequences to Nr database of NCBI to the terms of GO by use BLAST2GO (default parameters), and the calculated p-value goes through Bonferroni Correction, taking corrected p-value ≤ 0.05 as a threshold. GO terms fulfilling this condition are defined as significantly enriched GO terms in DTGs. For KEGG analysis, annotate to the KEGG database by BLAST (-p blastx-e 1e-5-m 8).

### Cluster analysis

Sets of genes showing a similar pattern of transcription were assumed to imply that they were functionally correlated. Transcription patterns were clustered using Cluster 3.0 [31] with Euclidean distances and the hierarchical cluster method of complete linkage clustering and Java Treeview software [32].

### Validation of DTG status using real-time RT-PCR

RNA extraction and first strand cDNAs Synthesis were carried out as described above. qRT-PCRs were performed using the Bio-Rad Real-time PCR Detection System (Bio-Rad, USA) based on the FastStart Universal SYBR Green Master mix (Roche, Basel, Switzerland), following the manufacturer’s instructions. Each 20 μl PCR contained 10 μl 2 × real-time SYBR Green I PCR Mix, 0.4 μl of each primer (sequences given in S1 Dataset) and an appropriate quantity of cDNA. The temperature cycling regime began with a pre-denaturation step (95°C / 30 s), followed by 45 cycles of 95°C / 15 s, 55°C / 10 s and 72°C / 10 s. We selected *Zm18S rRNA*, *ZmActin* and *ZmUBQ* housekeeping genes as a control to evaluate expression levels of selected genes. Three biological replicates were collected for each root type, and relative transcript abundance was calculated using the delta-delta Ct method [33].

### Measurement of soluble sugar content

Soluble sugar was measured according to Kong et al. [4]. In brief, to measure the soluble sugar content, frozen root material (0.3 g) was extracted with 10 mL H_2_O at 100°C for 10 min. The extracts were filtered and analyzed for soluble sugar content using the anthrone—sulphuric acid method. Briefly, 1 mL of the extract was mixed with 1 mL of H_2_O, 0.5 mL of anthrone reagent (1 g anthrone and 50 mL ethyl acetate) and 5 mL oil of vitriol, and then heated at 100°C for 1 min. After cooling, the mix was analysed by using UV spectrophotometry at 620 nm.

## Results and Discussion

### Differential growth of maize roots in response to salinity stress

**Fig 1:**
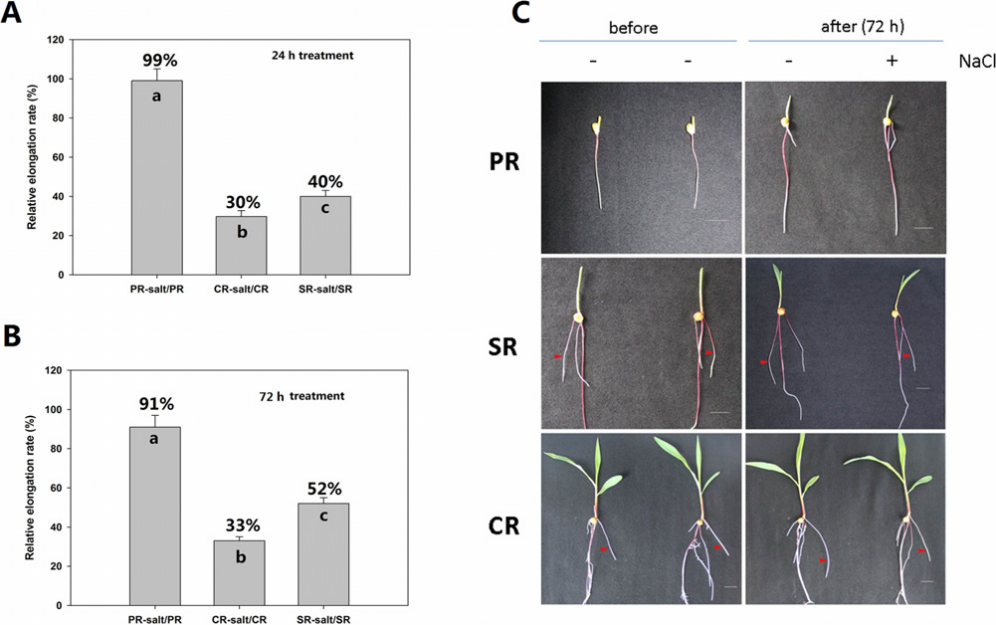
Sensitivity of the three types of maize roots to the salt treatment. (A) and (B) The relative elongation rate of the three types of maize roots after 24 h and 72 h exposure in 100 mM NaCl. Different letters represent significant difference at *p*< 0.05 (Duncan’s multiple range test; data are represented as mean ± SEs; three biological repeats). (C) The root morphology of PR, SR, CR with lengths of approximately 6 to 10 cm treated with 0 and 100 mM NaCl for72 h. Experiments were repeated three times with similar results. Bar = 2.0 cm.

Salinity stress is well known to inhibit root growth. The *A*. *thaliana* PR is less sensitive to salinity than the LR [18, 34]. Although the maize root as a whole is also sensitive [35], whether PR, SR and CR have similar or differential growth response to salinity stress is still unknown. When exposed to 100 mM NaCl for 24 h, the growth of the maize PR was less inhibited (1% reduction) than either the CR (70%) or the SR (60%) (Fig. 1A). Similar results were observed in the samples treated with 100 mM NaCl for 72 h (Fig. 1B,C). The assumption is that such a large difference must reflect distinct patterns of transcription, and therefore the differential metabolites in each root type.

### RNA-Seq analysis

Across the six RNA libraries (PR, PR-salt, CR, CR-salt, SR and SR-salt), once the low quality reads had been removed, over 5 x 10^7^ reads, representing 2.5 x 10^9^ nt, were acquired (Table 1). A high proportion of the reads were readily mapped to the maize reference genome sequence: 79.89% from PR, 78.87% from PR-salt, 77.80% from CR, 79.18% from CR-salt, 78.16% from SR, and 77.38% from SR-salt. A BLAST analysis assigned around two thirds of these sequences (respectively 66.12%, 66.52%, 65.47%, 67.04%, 64.85% and 64.84%) to known genes (Table 1).

**Table 1:** Summary of mapping result.

Sample ID	PR	PR-salt	CR	CR-salt	SR	SR-salt
**Total Reads**	8,278,219 (100.00%)	8,664,028 (100.00%)	8,641,655 (100.00%)	8,153,263 (100.00%)	8,265,737 (100.00%)	8,450,243 (100.00%)
**Total BasePairs**	405,632,731 (100.00%)	424,537,372 (100.00%)	423,441,095 (100.00%)	399,509,887 (100.00%)	405,021,113 (100.00%)	414,061,907 (100.00%)
**Total Mapped Reads (mapping to reference genes)**	5,473,192 (66.12%)	5,763,743 (66.52%)	5,658,068 (65.47%)	5,465,636 (67.04%)	5,360,742 (64.85%)	5,479,102 (64.84%)
**Total Unmapped Reads (mapping to reference genes)**	2,805,027 (33.88%)	2,900,285 (33.48%)	2,983,587 (34.53%)	2,687,627 (32.96%)	2,904,995 (35.15%)	2,971,141 (35.16%)
**Total Mapped Reads (mapping to reference genome)**	6,613,863 (79.89%)	6,833,217 (78.87%)	6,723,444 (77.80%)	6,455,367 (79.18%)	6,460,751 (78.16%)	6,539,064 (77.38%)
**Total Unmapped Reads (mapping to reference genome)**	1,664,356 (20.11%)	1,830,811 (21.13%)	1,918,211 (22.20%)	1,697,896 (20.82%)	1,804,986 (21.84%)	1,911,179 (22.62%)

### Identification of DTGs in the salinity stressed maize roots

**Fig 2:**
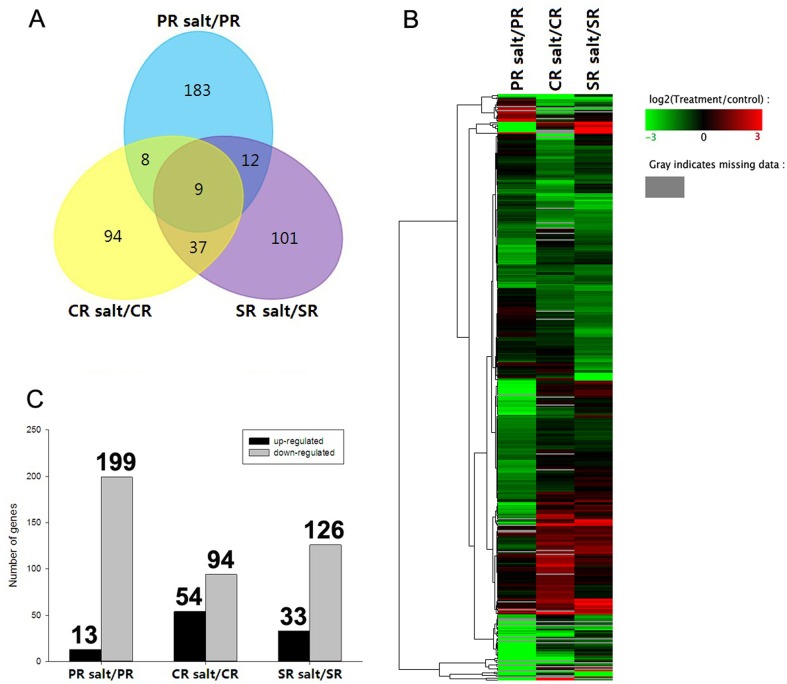
Transcription patterns of stress-regulated genes in the maize PR, CR and SR. (A) The total number of genes regulated by salinity stress in the three types of root. (B) Cluster analysis of salinity-regulated genes. Red indicates that the gene has a higher expression level in the salt-treated samples; green indicates that the gene has a lower expression in the salt-treated samples, and gray indicates that the gene has no expression in at least one sample. (C) The number of genes responding to salinity stress in each root type.

A total of 444 genes proved to be regulated by salinity stress (Fig. 2A, S2 Dataset). Of these, 212 (13 up- and 199 down-regulated) were identified in the PR under NaCl treatment, whereas 183 genes were specifically regulated in PR after salinity stress (Fig. 2C). Next, we compared the DTGs between CR with and without NaCl treatment; 148 genes showed differential transcription in CR under NaCl treatment, of which 54 showed up-regulated and 94 showed down-regulated (Fig. 2C). In addition, 94 genes were specifically regulated in CR after salinity stress. A total of 33 up-regulated genes and 126 down-regulated genes were found in SR after NaCl treatment (Fig. 2C), whereas 101 genes were specifically regulated in SR after salinity stress. After cluster analyses, as shown in Fig. 2B, PR showed distinct transcription profiles of DTGs compared with SR and CR, which had similar transcription profiles of DTGs after salinity stress. In conclusion, PR showed the largest total and specifically regulated DTGs after salinity stress (S2 Dataset). Furthermore, a relatively larger portion of DTGs were down-regulated in PR (93.9%) than in CR (63.5%) and SR (79.2%) after salinity stress (Fig. 2C). These results suggested that the differential transcription trends of the DTGs in PR, CR, and SR may contribute to the regulatory mechanism of salinity sensitivity in different roots (S3 Dataset).

**Fig 3:**
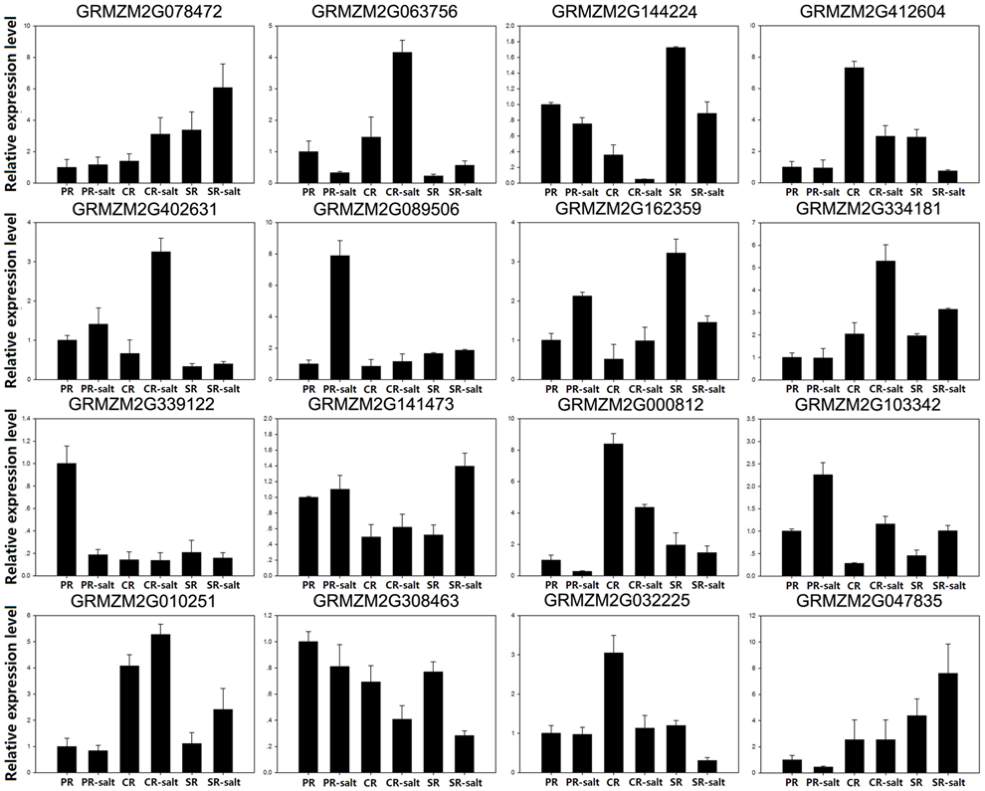
qRT-PCR validation of differential transcription identified by RNA-Seq. Each column represents an average of three replicates, and bars indicate SEs.

A random sub-set of 16 of the 444 DTGs was subjected to real-time quantitative PCR (qRT-PCR) to validate the RNA-Seq data. As shown in Fig. 2B, Fig. 3 and S1 Fig., the qRT-PCR output of about 70 percent of 16 genes confirmed the RNA-Seq based identification of DTGs. For example, the expression of GRMZM2G339122 (EXP1) was down-regulated in PR, but has no change in CR and SR after salt treatment, and GRMZM2G010251 (NRT2:1) was found to be down-regulated in PR but up-regulated in CR and SR. However, some genes did not correspond with RNA-Seq data. For example, our qRT-PCR results showed that GRMZM2G089506 (Eukaryotic aspartyl protease family protein) was up-regulated in PR, but down-regulated in PR in RNA-Seq data.

### Functional classification of DTGs under salinity stress

**Fig 4:**
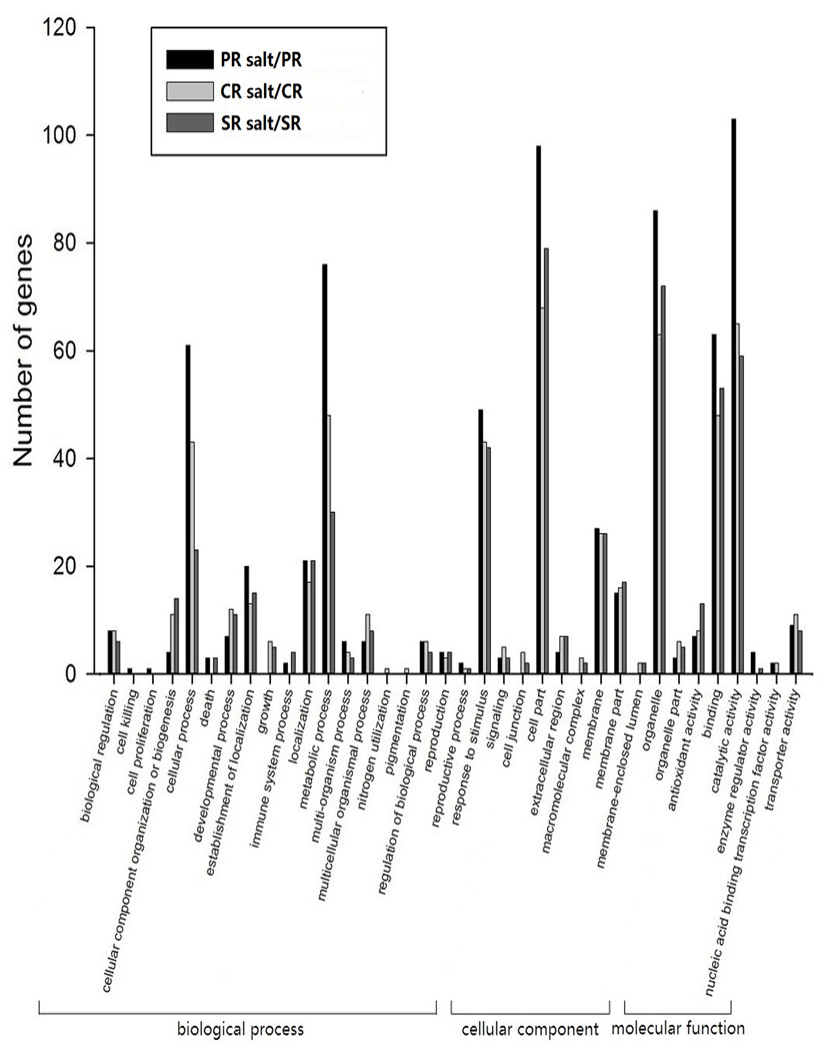
Functional classification (GO) of salinity-regulated genes in the maize PR, CR and SR.

The likely function of the set of DTGs was explored using the GO classification system (Fig. 4). With respect to biological process, many of the genes fell into the categories cellular process, establishment of localization and metabolic processes; with respect to the cellular component, the main categories represented were response to stimulus, cell part and membrane; and with respect to molecular function, the key categories were binding, catalytic activity and organelle. DTGs were more abundant in the PR than in the CR or SR after salinity stress in all three categories. The outcome indicated that salinity stress induced antioxidation and transcription factor activity, which, in turn, generated the synthesis of solutes such as proline, trehalose, mannitol and lycine, as maybe a common mechanism underlying salinity tolerance in maize roots, especially in PR [36–38].

**Fig 5:**
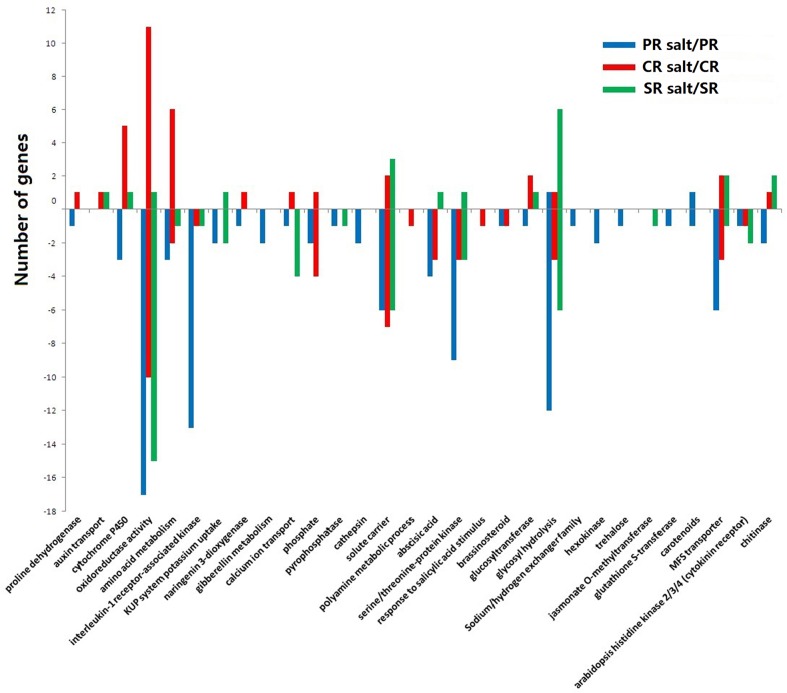
Pathway analysis (KEGG) of salinity-regulated genes in the maize PR, CR and SR.

When the DTGs were further subjected to KEGG analysis, the major pathways regulated by salinity stress were revealed to oxidoreductase, glycosyl hydrolysis, receptor-assotiated kinase and ABA signaling (Fig. 5 and S4 Dataset), a result consistent with what has been reported elsewhere [18, 39–43].

### DTGs related to oxidoreductase activity

**Fig 6:**
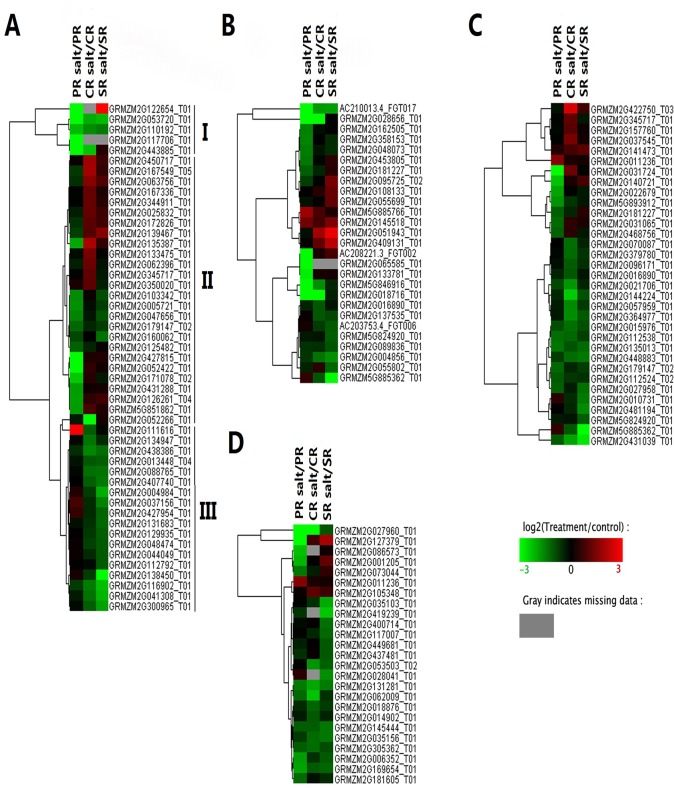
Categories of salinity-regulated genes in the maize PR, CR and SR. Genes encoding (A) oxidoreductases, (B) glycosyl hydrolases, (C) phytohormone synthesis and (D) transcription factors. Red indicates that the gene has a higher expression level in the salt-treated samples; green indicates that the gene has a lower expression in the salt-treated samples, and gray indicates that the gene has no expression in at least one sample.

Salinity stress induces both osmotic and ionic stress. The former is perceived rapidly by the plant, and soon inhibits root growth, water uptake and cell expansion. A common cellular manifestation of this stress is the production of reactive oxygen species (ROS), which damage cell growth through their degradative effect on proteins, lipids and DNA [39, 44]. In order to mitiagate this damage, plants have evolved a range of antioxidative enzymes and non-enzymic compounds [44]. Overexpression of a Mn-SOD (Superoxide Dismutase) in transgenic *Arabidopsis* plants showed increased salt tolerance; over-expressing *OsAPXa* or *OsAPXb* (Ascorbate Peroxidase) exhibited increased salt tolerance in transgenic *Arabidopsis* plants due to higher APX, low H_2_O_2_ and MDA (Malondialdehyde) content. Thus the constitutive expression of a superoxide dismutase or certain peroxidases was able to enhance the level of salinity tolerance shown by *A*. *thaliana* [45]. Here, the transcription of the genes GRMZM2G427815 (POX52) and GRMZM2G138450 (POX), identified as encoding members of a peroxidase superfamily, contrasted between the PR and the CR and SR in response to salinity stress (Fig. 6A). In all, 49 genes related to oxidoreductase activity were up- or down-regulated in response to salinity stress (Fig. 2 and Fig. 6A). A cluster analysis recognized three types (I, II and III) among these genes. Type I members, typified by GRMZM2G053720 (POX), GRMZM2G110192 (nine-cis-epoxycarotenoid dioxygenase 4, NCED4) and GRMZM2G117706 (POX), were down-regulated by salinity stress in all three root types. The response of type II genes was a fall in transcript abundance in the PR, but an increase in the CR and SR; an example is GRMZM2G052422, a maize homolog of *A*. *thaliana AtACO4* (which encodes an ACC oxidase) (Fig. 6A). It has been previously shown that ethylene and its precursor 1-aminocyclopropane-1-carboxylic acid (ACC) inhibit root cell elongation in *A*. *thaliana* roots [46]. It is possible that salt stress stimulates a much stronger ethylene signaling effect on CR and SR than PR growth inhibition. Finally, the type III genes displayed down-regulation in the CR and SR, but slight up-regulation in the PR. GRMZM2G111616 is one example of type III genes which belongs to formate dehydrogenase family. These results suggested that genes involved in redox reaction play an important role in maize root system in response to salt stress, especially in PR.

### DTGs related to glycosyl hydrolysis

Glycosyl hydrolases have been classified into more than 45 families [47, 48], several of which have been implicated in the abiotic stress response. An example is *AtBG1*, an *A*. *thaliana* β-glucosidase, which is important in the drought stress response [49] and is negatively regulated by salinity in sweet almond [50]. *CaChi2*, a chitinase gene from pepper, is induced by ABA, NaCl and drought [51]. Here, 27 genes involved in glycosyl hydrolysis were identified as being regulated by salinity (Fig. 6B), with the majority contrasting between the PR and the CR/SR with respect to their transcript accumulation. Both GRMZM2G051943_T01 and GRMZM2G133781_T01 (both encoding chitinases) were up-regulated by salinity in the CR and SR, but not in the PR. The gene GRMZM2G065585_T01 (glycosyl hydrolysis family 17) was down-regulated in the PR, but in neither the CR nor the SR, while At4g16260, an *A*. *thaliana* homolog of GRMZM2G065585_T01 (Glycosyl hydrolase superfamily protein), is up-regulated by salinity stress [52]. GRMZM2G055802_T01 (BBTI13) and AC208221.3_FGT002 (BBTI2), homologs of, respectively, the rice genes *OsBBTI13* and *OsBBTI2*, were differentially transcribed between the PR and the CR in salinity stressed plants (Fig. 6B). *OsBBTI13* is a component of the abotic stress response of rice [53]. Our results indicated that genes involved in glycosyl hydrolasis played an important role in different maize roots in response to salt stress.

### DTGs related to phytohormone synthesis

Plant growth and development relies on the interplay of a suite of phytohormones. The synthesis of abscisic acid (ABA) is an early response to many abiotic stresses [18, 41]. ABA metabolism is under the control of the cytochrome P450 type enzyme CYP707A; in the absence of this enzyme, the level of tolerance to drought stress is enhanced [54], which suggested that *CYP*707A plays a negative role in abiotic stress through determining the endogenous level of ABA. Here, the KEGG analysis showed that, in the transcriptome of salinity-stressed plants, genes encoding cytochrome P450 type enzymes were under-represented among the PR DTGs, but over-represented among the CR and SR ones (Fig. 5 and S3 Dataset). ABA signaling in the endodermis is known to represent an important mechanism underlying the differential response of the *A*. *thaliana* PR and LR to salinity [18]. The transcription profiles of genes which are related to synthesis of phytohormones and signaling, such as GRMZM2G096171 (type-A response regulator, involved in cytokinin signaling), GRMZM2G031724 (GA2OX1, involved in gibberellin signaling) and GRMZM2G031065 (gibberellin receptor GID1L2, involved in gibberellin signaling), contrasted between the PR and the CR/SR (Fig. 6C), while those of other genes, such as GRMZM2G140721 (CRK25, involved in brassinosteroid signaling) responded differentially to salinity stress in each of the three root types (Fig. 6C).

### Differentially transcribed TFs

It has been established that transcription factors (TFs) including NAC, WRKY, MYB, AP2, bHLH, and C2H2 like zinc finger, HSF, and bZIP play central roles in plant abiotic stress responses by regulating downstream genes via specific binding to *cis*-acting elements in the promoters of target genes. Overexpression of *Arabidopsis* SNAC-A genes such as *RD26* and *ATAF1*, and rice SNAC-A genes such as *OsNAC6* and *OsNAC5* can improve drought and salinity tolerance [55, 56]; *OsWRKY30*, which is induced by PEG, NaCl and ABA treatment, confers the osmotic stress tolerance in transgenic *Arabidopsis* [57]; Constitutively overexpressing any one of *DREB*1A*/B/C* genes which belongs to AP2/ERF family display significantly improved tolerance to freezing, drought and high salinity in transgenic *Arabidopsis* [58, 59]. Our transcriptome analysis revealed that 25 TFs were induced or repressed in response to salt stress, and these genes were identified from 13 different families including C2H2 like zinc finger, NAC, AP2/ERF, LBD, C3H zinc finger, HSF, MYB, WRKY, bHLH, TALE, DBB, as well as Trihelix and RAV (Fig. 6D) and our results showed that most of detected TFs were down-regulated after salt stress in all three different maize roots. Previous studies showed that overexpression of *TaNAC2*, *OsNAC6* and *OsNAC5* enhanced tolerances to drought, salt, and freezing stresses [6, 55, 56]. GRMZM2G127379 (NAC family) was up-regulated by salinity in the CR and SR, but down-regulated in the PR, as well as GRMZM2G001205 (C2H2 like zinc finger). These trends may be due to the longer treatment period (24 h). However, GRMZM2G011236 (ERF family) showed an opposite expression pattern between PR and CR, SR (Fig. 6D). Taken together, these results reveal that the differential expression trends of these TFs, especially GRMZM2G127379 (NAC family), in PR, SR, and CR may contribute to the regulatory mechanism of salinity sensitivity in different roots.

### PR accumulate more soluble sugar after salt stress

It has been determined previously that the accumulation of soluble sugar in plants is correlated with increased tolerance to salt stress [4]. To test if the higher tolerance of PRs to salt stress is attributed to the higher level of soluble sugars, we measured the soluble sugar contents in PR, SR and CR. As shown in S2 Fig., PR accumulated a higher level of soluble sugar under NaCl treatment even under normal conditions, which is consistent with the higher tolerance of PR to salt stress.

## Conclusion

The overall finding from these experiments was that the three maize root types showed a distinct response to salinity stress, and that the PR was more tolerant of the stress than was either the CR or the SR. The RNA-Seq analysis identified over 400 genes as being differentially transcribed in response to salinity stress, and the functional analysis of these DTGs suggested that the most important genes involved in the stress response were, apart from various upstream TFs, those dealing with ROS, with glycosyl hydrolysis, with hormone signal perception and transduction, and with the synthesis of compatible solutes such as soluble sugars. The study revealed that the PR, SR, and CR each had its own distinct transcriptional profile, which might underlie the differential growth of each root type in the face of environmental challenge.

## Supporting Information

S1 Dataset PCR primers used in this study. (XLSX)

S2 Dataset Differentially Transcribed Genes (DTGs) (log2 Ratio ≥ 1, FDR ≤ 0.001). (XLSX)

S3 Dataset Genes regulated specifically in each root type after NaCl treatment (log_2_ Ratio ≥ 1, FDR ≤ 0.001). (XLSX)

S4 Dataset KEGG pathway enrichment analysis of DTGs. A: KEGG pathway enrichment analysis of DTGs in PR salt/PR. B: KEGG pathway enrichment analysis of DTGs in CR salt/CR. C: KEGG pathway enrichment analysis of DTGs in SR salt/SR.
(XLSX)

S1 Fig Validation of the differential transcription of 16 of the genes identified by RNA Seq, based on qRT-PCR, which was made by SigmaPlot 11.0 program. **: The transcript abundance in the nontreated samples and the salt treatment samples differs significantly at *p*< 0.01.
(TIF)

S2 Fig Soluble sugar contents in PR, SR and CR under salt stress. Soluble sugar content in each root type treated with 100 mM NaCl for 24 h. Each column represents an average of three replicates and bars indicate SDs. ** and * indicate significant differences in comparison with PR at *P* < 0.01 and *P* < 0.05, respectively.
(TIF)
