# Supplementary figures and images for: Comparative Transcriptome Profiling of the Maize Primary, Crown and Seminal Root in Response to Salinity Stress
Source: PLoS One. 2015 Mar 24;10(3):e0121222. doi: 10.1371/journal.pone.0121222 (PMC4372355; doi:10.1371/journal.pone.0121222)

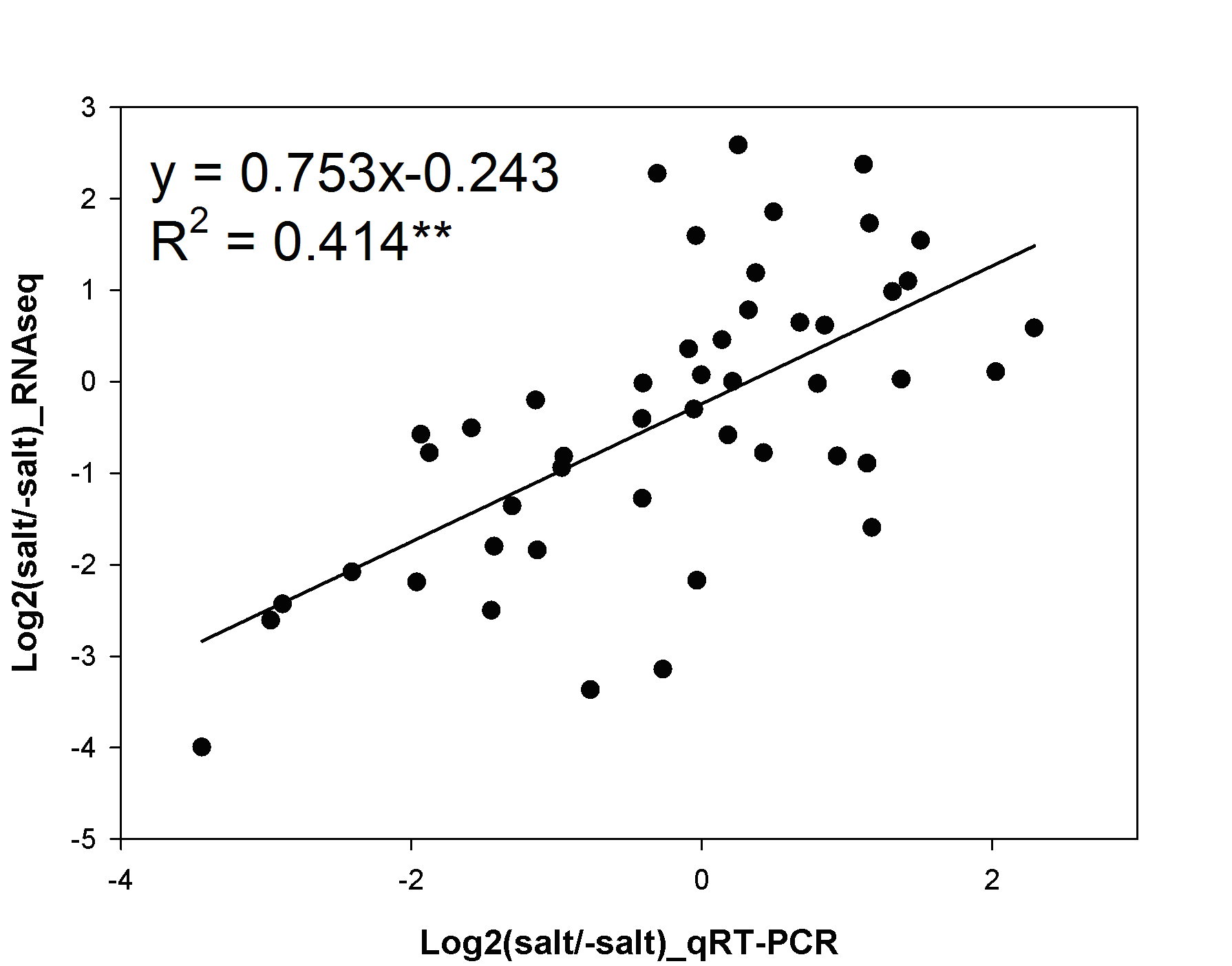

Supplement: S1 Fig — **: The transcript abundance in the nontreated samples and the salt treatment samples differs significantly at p< 0.01. (TIF) [file pone.0121222.s005.tif]

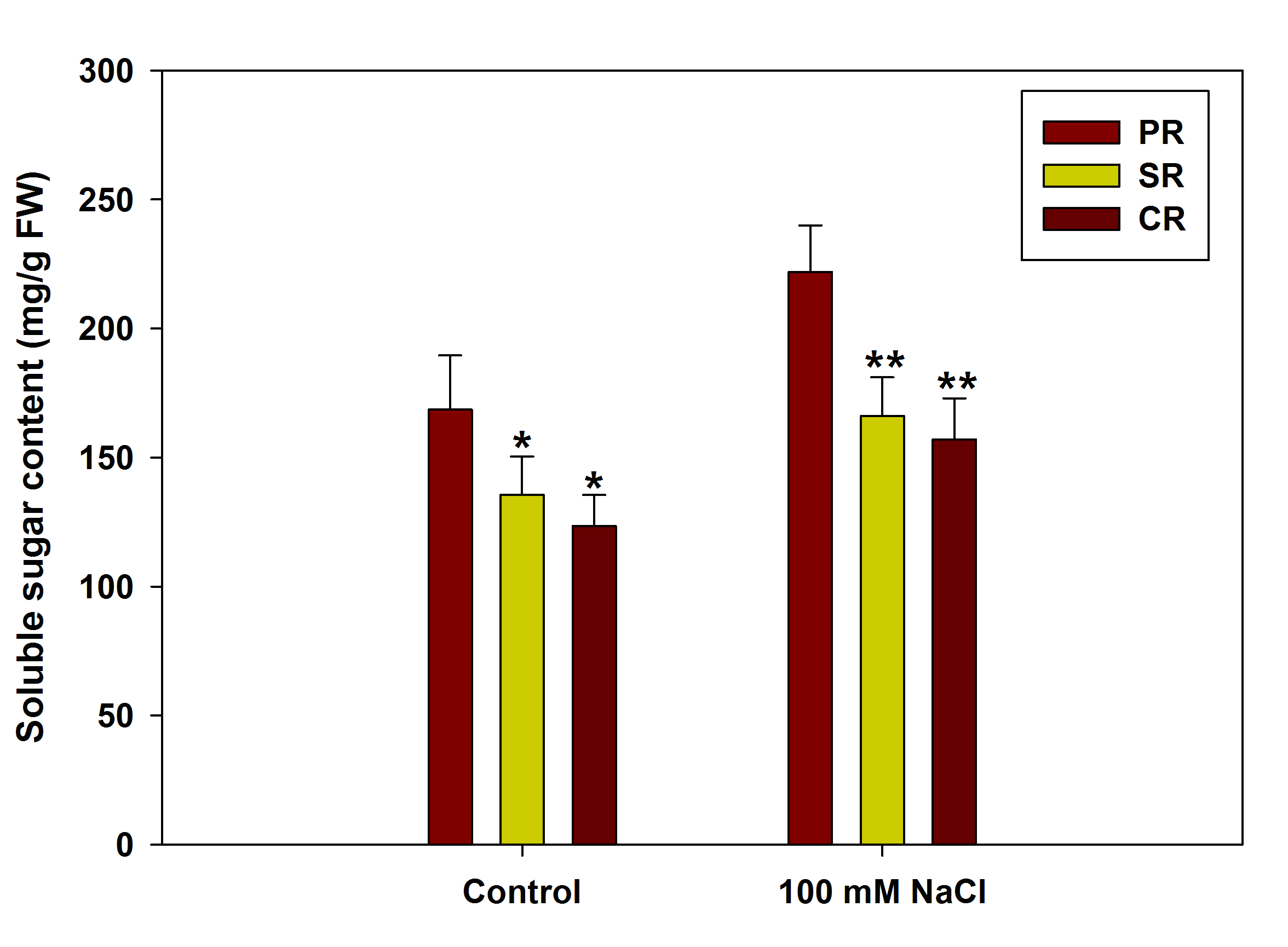

Supplement: S2 Fig — Soluble sugar content in each root type treated with 100 mM NaCl for 24 h. Each column represents an average of three replicates and bars indicate SDs. ** and * indicate significant differences in comparison with PR at P < 0.01 and P < 0.05, respectively. (TIF) [file pone.0121222.s006.tif]
